# Perceptions of occupational therapists and physiotherapists of early intervention therapy services for infants with or at risk of cerebral palsy: A qualitative interview study

**DOI:** 10.1177/18758894251385505

**Published:** 2025-10-31

**Authors:** Helle Sneftrup Poulsen, Alice Ørts Hansen, Lone Walentin Laulund, Charlotte Ytterberg, Lisbeth Rosenbek Minet

**Affiliations:** 1 HCA Childreńs Hospital, 11286 Odense University Hospital (OUH), Odense, Denmark; 2 HCA Research Unit, Department of Clinical Research, OUH and University of Southern Denmark (SDU), Odense, Denmark; 3 Geriatric Research Unit, Department of Clinical Research, OUH and SDU, Odense, Denmark; 4 Research Center for Person-centered Rehabilitation, 72736 SDU, Odense, Denmark; 5 Research unit of Orthopedic Surgery and Traumatology, Department of Clinical Research, OUH and SDU, Odense, Denmark; 6 Neurobiology, Care Sciences and Society, Karolinska Institutet, Stockholm, Sweden; 7 Women's Health and Allied Health Professionals Theme, Karolinska University Hospital, Stockholm, Sweden; 8 FaCe Family Focused Healthcare Research Center, SDU, Odense, Denmark

**Keywords:** cerebral palsy, early intervention, occupational therapy, physical therapy, evidence-based practice

## Abstract

**Purpose:**

This study aimed to explore therapists’ experiences of providing early intervention therapy services for infants with or at risk of cerebral palsy (CP), and, in particular, therapy services that promote infants’ hand function.

**Methods:**

Eleven semi-structured small group interviews and one individual interview were conducted with 26 therapists working with infants, either in hospital or in the municipality. Interview transcripts were analyzed using qualitative content analysis.

**Results:**

Providing early intervention therapy services for infants with or at risk of CP was influenced by factors such as inadequate coordination and communication between hospital and municipalities, varying use of motor assessment tools to detect risk of CP, more focus on gross motor function than hand function in early age, impact of usual clinical practice, requirements for acting in a variable therapeutic role when providing guidance for parents, and challenges obtaining therapeutic training and specialization in a decentralized organization.

**Conclusion:**

Challenges caused by a complex practice and a high level of requirement for therapeutic skill may have an impact on evidence-based practice and need to be addressed when incorporating new research knowledge about early CP-specific interventions into a family-centered clinical practice.

## Introduction

Cerebral palsy (CP) is a disorder of the development of movement and posture,^1^ and it is a common cause of physical disability in children.^2^ In high-income countries, the overall CP birth prevalence is estimated to be 1.6 per 1000 live births.^3^ In children with CP, manual ability and bimanual performance are strongly related to independence in self-care^4^ and participation in everyday activities.^5^ Therefore, promoting hand function in these children is a highly important treatment goal^6^ in the services provided by occupational therapists (OTs) and physiotherapists (PTs).

The diagnosis of CP is based on both clinical and neurological signs, and often the diagnosis cannot be made with certainty in the first year of life.^7^ Therefore, to enable an early specific intervention and family support during infancy, when neuroplasticity is high,^8,9^ early detection of risk of CP is recommended.^7,10^ Strong evidence supports using the standardized motor tool General Movements Assessment (GMA), combined with neuroimaging and clinical history, to predict risk of CP before five months corrected age.^7^ If an infant without a medical history indicating risk of CP shows clinical signs of CP, prompt referral for diagnostic assessment is recommended.^11^ In terms of hand use, risk of unilateral CP can be predicted from the age of 3.5 months, by using the tool Hand Assessment for Infants (HAI).^12^

Regarding early specific interventions to improve motor skills development in infants with or at risk of CP, recent research evidence supports task-specific, and goal directed interventions that include child-initiated movement, enriched environments, repetition, and high intensity.^2,13^ An example of an early CP-specific intervention is constraint induced movement therapy for infants.^14^ However, a recent survey suggests that PTs and OTs do not sufficiently incorporate early CP-specific interventions into practice.^15^

In addition, using a family-centered approach in service delivery is considered best practice in early intervention and pediatric rehabilitation.^16^ Family-centered practice (FCP) emphasizes families as experts, informed decision making, and the family-provider partnership,^17^ which includes the aspect of collaborative goal setting.^18^ However, despite a theoretical foundation and evidence supporting FCP, implementation into practice remains challenging.^17,19^

The translation of new research evidence into clinical practice is known to be a challenge that takes time, which is problematic, as evidence-based practice (EBP) is essential to improving infants’ outcomes.^20^ EBP implies that research evidence is integrated with knowledge from therapists’ experiences and families’ values and preferences to inform decision making^21^ for infants’ early intervention. Several studies have investigated therapists’ experiences of providing early intervention for children, in general. Challenges reported include lack of consistency in how FCP is defined and implemented,^22^ the need for training of professionals, inadequate collaboration and communication between professionals, and managing family dynamics, attitudes or barriers related to language, socioeconomic status and culture.^19,22,23^ To the authors’ knowledge, only one survey has examined the experiences of therapists providing early intervention therapy services for infants with or at risk of CP.^24^ This study reported similar challenges including professionals’ lack of knowledge or qualifications, inadequate collaboration and communication, and challenges in meeting complex family needs.^24^ Therefore, a more in-depth understanding of the therapist's perspective on providing this specific service is needed to uncover possible challenges related to an early family-centered and EBP.

In a public healthcare system like the Danish one, the services provided by OTs and PTs are free of charge for families. Hospital-based therapists assess infants’ motor development in case of concern. If a need for rehabilitation is identified, the infants are referred to PTs or OTs in the municipality where the family lives as a part of a formal discharge process intended to ensure continuity of rehabilitation. Intervention typically takes place at home or in daycare after 48 weeks of maternity leave. After maternity leave, parents can apply for leave to take care of the infant's training needs, including payment of private service providers engaged by the parents. All children diagnosed with CP are included in the national interdisciplinary and cross-sectoral Cerebral Palsy Follow-up Program (CPOP).^25^

The aim of this study was to explore therapists’ experiences of providing early intervention therapy services for infants with or at risk of CP, particularly in relation to promoting infant hand function.

## Materials and methods

### Design

This study employed a qualitative design, which is suitable when the aim is to provide in-depth insights into a phenomenon as experienced by individuals in their own context.^26^

### Participants & setting

The study was carried out in the region of Southern Denmark. The participants were recruited either from the four hospitals in the region or from eight out of 22 municipalities. Using a purposeful sampling strategy, the eight municipalities were selected with a focus on variation in their geographic context (whether it is situated in a rural or urban setting) and size (number of inhabitants and number of therapists working with children), so that small, medium, and large municipalities were represented. Included participants were OTs and PTs working with infants with or at risk of CP. Workplaces with several possible participants selected the participants after being approached by the first author (HSP).

The research was carried out in accordance with the principles and standards outlined in the Danish Code of Research Integrity.^27^ The study was approved by the Danish Data Protection Agency, no. 19/29790. Participants were informed of their voluntary participation and right to withdraw anytime, with written consent obtained.

### Data collection

Data were collected through eleven small group interviews (2–3 participants from the same workplace) and one individual interview using a semi-structured interview guide with open-ended questions. The guide covered five content areas: transition between hospital and municipality, the early intervention being offered, hand function in early intervention, parent involvement and education, and the therapist's role and skills. Twenty-six participants were included: 17 PTs and nine OTs, all women. Nine were hospital-based and 17 worked in municipalities. Their experience of working with children ranged from three to 40 years, with an average of 21 years. The interviews were conducted by the first author (HSP) between February 2022 and March 2023. The interviews were recorded, lasted 49–63 min, and were transcribed verbatim.

### Data analysis

**Figure 1.**
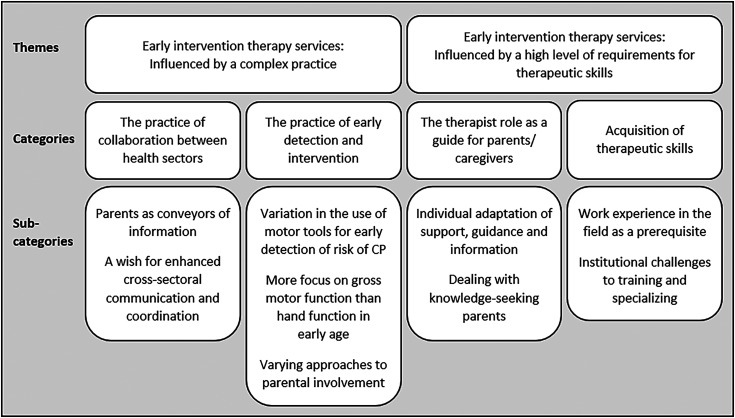
Sub-categories, categories and themes revealed during the analysis.

Data were analyzed using qualitative content analysis, with interview transcriptions structured, coded, and analyzed according to the approach described by Graneheim and Lundman.^28^ First, the material was read several times, to gain an overall impression of the data. Then, the meaning units corresponding to the aim were identified and condensed, without losing their meaning, and labeled with a code. After that, the codes were sorted and abstracted based on similarities and differences – first into sub-categories and then into categories. This was an iterative process that ended with the formulation of four categories on a descriptive level and, thus, an expression of the manifest content across the material that remained close to the text. At this stage of the analysis, it became clear that the data allowed for a higher level of interpretation. Thus, through condensed meanings units, codes and categories, the underlying meanings were abstracted into two themes, on an interpretative level. An example of the analysis process is shown in Table 1. Sub-categories, categories and themes are presented in Figure 1.

**Table 1.** Example of meaning unit, condensed meaning unit, code, interpretation and theme.

Meanings unit	Condensed meanings unit (descriptive)	Code	Sub-category	Category	Interpretation of the underlying meaning	Theme
OT1: … but we also deal with parents who search the internet, so we are dealing with different parents than just 10 years ago. PT: Yes. OT1: They are much more informed, sometimes a little too well informed [about possible severity of CP], where we almost have to slow down a bit and say, you know what, it doesńt have to be that bad. (Municipality 18)	It is a different group of parents than 10 years ago. Parents search the internet and are much more informed, sometimes too well informed.	Therapists must be able to support information-seeking parents and to reassure them.	Dealing with knowledge-seeking parents	The therapist's role as a guide for parents/ caregivers	Therapists must be able to accommodate parents’ fear/ concern and deal with parents searching the internet for information on CP.	Providing early intervention: Influenced by high level of requirement for therapeutic skills

CP: cerebral palsy; OT: occupational therapist.

The first author (HSP) conducted the main analysis. To ensure trustworthiness, two authors (AOH, LMR) also read and coded the material, and, through discussion and reflection (HSP, AOH, LMR), agreement was reached on tentative categories and themes. Then, the analysis was validated through repeated discussions until consensus was reached (HSP, AOH, CY, LMR). Two of the authors (LRM, CY) have expertise in qualitative research. All authors agreed on the final abstraction of sub-categories, categories and themes. The credibility of the result was ensured by selected quotations from the interviews. The translation of the quotations to English was validated by a professional translator.

## Results

Overall, the qualitative content analysis revealed two overall themes with four corresponding categories and nine subcategories about therapists’ experiences of providing early therapy services to infants with or at risk of CP (Figure 1).

### Theme 1. Early intervention therapy services: influenced by a complex practice

The first theme abstracted from participants’ experiences concerned the impact of the complexity in practice on the early intervention therapy service provided.

#### The practice of collaboration between health sectors

*Parents as conveyors of information.* A challenge described by all participants concerned the exchange of information between health organizations, where parents had to become conveyors of information. Most participants from the municipality experienced a lack of transferred written information from the hospital about medical history and treatment when an infant was referred to follow-up. Since they were prohibited by the General Data Protection Regulation to access infants’ medical records, they sometimes had to ask parents to obtain the necessary information before they could provide the appropriate treatment to the infant. The participants perceived this as placing a burden on already stressed parents. Two participants stated it this way:

*PT:* *…* *that's when we have to say to the parents, wouldn’t you like to request your child's medical record, because you are allowed to do that as parents* *…* *because that's really important knowledge to have, for me as a professional, who is going to help you, going forward* *….*

*Interviewer: So, the parents are put in a position where they are the ones who have to collect the information?*

*OT & PT: Yes.*

*PT: And that is actually a huge pressure for the parents. (Municipality 14)*

In addition, participants experienced a lack of routine for exchanging information when an infant who received therapy in the municipality went for a checkup at the hospital. Therefore, they sometimes had to ask parents to orally pass on professional information between sectors.

*A wish for enhanced cross-sectoral communication and coordination.* Based on participants’ experiences, cross-sectoral home visits, checkups or meetings were a good way to ensure sufficient communication and a coordinated therapeutic service, and they suggested that this practice be used more. Particularly, cross-sectoral meetings where parents heard that all health professionals agreed were perceived as important to avoid parents receiving conflicting information that could hinder an optimal early intervention. For example, if the hospital doctor was not completely up to date on therapeutic interventions, this could lead to a less optimal intervention for the infant, as the participants experienced that parents tended to follow the doctor's recommendations. One participant stated it this way:

*PT: What we do experience sometimes is that, when the infant has been at the hospital, they [the parents] still get the same old [recommendation regarding stretching], and that is the one they follow. In any case … when I come out for a home visit and say, “don’t spend all that energy on those passive stretching exercises” … I often experience that the parents then say, but the hospital [doctor] says I should do these exercises. (Municipality 7)*

#### The practice of early detection and intervention

*Variation in the use of motor tools for early detection of risk of CP.* Participants, PTs in particular, experienced that infants were referred to them when there was a concern regarding an infant's motor development. Hospital participants described that the therapists’ primary task was to assess infants’ motor function. They expressed having knowledge about motor tools used for early identification of risk for CP, such as GMA and HAI. However, according to participants’ experiences, the use of these tools varied, and they were not used as part of an interdisciplinary standard routine for early identification of risk of CP. At two hospitals, motor screening was performed in a non-standardized way, as here stated by two participants:

*OT: Before they [the families] go home, I often do a motor screening on them [the infants]. I don’t have an assessment tool or anything, that is –*

*PT: We don’t have General Movement [Assessment tool].*

*OT: No, we don’t have General Movement. Neither do we have that TIMP [assessment tool], because for too long I have tried to get started with it, and neither do we use, eh –*

*PT: AIMS [assessment tool]. (Hospital 4)*

Most municipal participants had experienced detecting motor signs of CP in infants who did not have a medical history that could indicate risk of CP. Participants stated that these infants typically were identified at an age when signs of CP started to appear more clearly in their motor development and that they then initiated a referral to the hospital for diagnostic evaluation.

*More focus on gross motor function than hand function in early age.* Participants described that, in early age, the focus was mainly on promoting gross motor skills rather than hand motor skills. Although they focused on hand use interventions, some participants experienced that the parents’ attention was directed towards their infant's gross motor skills. Parents’ focus on hand function often came later in the child's early life, when it was harder to change the child's habits. One participant expressed that this could lead to grief in the parents:

*PT: I don’t think it is that [the hand function], that is the greatest focus for the family, and I also experience that that is what they sometimes find hardest to work with … the motivation to do something, it, it, it comes a little too late with regard to including the hand* *… If [the infant] gets to a certain age, well, then it doesn’t get any easier* *… I don’t know if it becomes a greater grief, but in a way it becomes a grief, because it kind of sneaks in. (Municipality 9)*

Mostly, participants described that interventions targeting hand function were addressed by an OT, and were offered later in a child's life, when the expectations of a child's hand motor skills increased.

*Varying approaches to parental involvement.* Throughout the interviews, participants described the importance of early therapy intervention being integrated into everyday activities, i.e. into family routines. Thus, a central part of the therapeutic practice was to involve parents as those who carried out therapy on a daily basis.

The approach to involvement of parents varied in practice. Most participants experienced no need for shared goal setting at an early age. They described that the infants’ motor developmental milestones provided the focus for the intervention, which they communicated to the parents. They felt it was just as important to give parents an understanding of why a specific activity or exercise made sense for their infant's development. In the hospital, it was considered too early for shared goal setting and that it should be introduced by the therapists in the municipality:

*PT: In relation to those [infants] that I see, we don’t talk about goal setting. It is something that I think they [the parents] are introduced to in the municipality … I also think we are in a phase that is so early that I can imagine the parents saying we don’t know what needs to be done. (Hospital 19)*

However, some participants from the municipality described that they did involve parents in goal setting. The goal setting process was sometimes challenging, such as formulating concrete, short-term goals. Some participants stated that their involvement of parents in goal setting had increased over the years, and they experienced that the involvement depended on the skills of the individual therapist.

*PT2:* *…* *We have improved our ability to get the parents involved [in goal setting].*

*PT1: We have got better, I think so too.*

*PT2: It is possible that one could be better at it, especially regarding this target group … after all, it is up to each therapist, how good one is at getting the parents involved in it, right? (Municipality 23)*

### Theme 2. Early intervention therapy services: influenced by a high level of requirement for therapeutic skills

The second theme concerned the impact of the high level of requirement for multiple therapeutic skills on the early intervention therapy service provided.

#### The therapist role as a guide for parents/caregivers

*Individual adaptation of guidance, information, and support.* Participants described that their guidance and support to parents depended on their perception of the parents’ resources, ability to translate the guidance into practice, attitudes to training in daily activities and ethnic background. They also described that they had to balance what the infant and the parents could handle in a way that supported the parent-infant relationship.

Participants’ guidance also depended on their own perceptions of where the parents were in their crisis and grief related to not having a healthy infant, and how they coped with it. Sometimes, participants experienced difficulty initiating parent-delivered therapy, which was perceived as a dilemma in relation to reaching a high intensity of therapy. A participant stated that there were situations in which they had to be able to handle parents being sad and crying and still try to find a way to gently start an intervention for the infant. At other times, participants experienced parents who performed an extensive amount of treatment for their child. They expressed that they then had to support parents in prioritizing their efforts, which was perceived as important to avoid parents becoming distanced in the relationship with their infant, here expressed by two participants:

*PT: The dilemma becomes just that* *…* *We know as therapists how essential it is, that this [training] is done many times, right. So, it becomes such an integrated part of the way one handles the infant* *…* *But when one [the therapist] goes to the family home and meets the parents and, and the infant at the beginning, it is wildly dependent on where the parents are in their shock reaction* *…* *So, you can visit them at home with the best and greatest intentions on behalf of the infant about starting [training], but if the parents are in a position* *…* *where they cannot be guided in any of this training, then it has to be given bit by bit.*

*OT: Yes and* *…* *[other parents], they just want to do something, fix something, do something, so, in that case, they ask for more. But, where we then have to do this prioritization regarding how much of an effort must be put into, eh, because it can become such a distancing for the parents regarding actually not really seeing the child and engaging with them just in –*

*PT: Yes. (Municipality 14)*

*Dealing with knowledge-seeking parents.* Participants experienced that parents search for information on the internet, which gives rise to diverse questions that they sometimes felt were demanding to deal with. Participants sometimes needed to encourage parents to rely less on information they had found, e.g. if they had read about children who were more severely affected by CP than their own infant (see quotation, Table 1). Participants expressed that, as therapists, they also had to respond to alternative methods of treatment that the parents asked about or had chosen to practice privately. The parental search for alternative methods was perceived by participants as an expression of, for example, a search for hope and a wish to feel they had done all in their power to help their infant. One participant stated it this way:

*PT1: The thing about seeking knowledge and trying to buy something more and something else, in the hope of being able to help your infant, of course. (Hospital 10)*

In relation to parents’ search for the best treatment methods, participants experienced an increasing need to be able to explain the evidence that supported their own practice, e.g. by referring to guidelines on the Danish Health Authority's website, as they felt the parents then found them more credible.

#### Acquisition of therapeutic skills

*Work experience in the field as a prerequisite.* Throughout the interviews, providing early intervention therapy services was viewed as a complex task that required experience-based knowledge and skills. Participants expressed that therapists without experience in this field needed to build up experience through side-by-side training and supervision by experienced colleagues, especially when therapists had to work alone in the family home.

Participants described that therapists had to gain clinical expertise in assessing infants’ sensorimotor development and training needs, including being able to analyze infants’ movement patterns and to motivate infants to participate in play appropriate to their developmental age. They also had to be able to sense infants’ response to stimulation with their hands as a basis for guidance or intervention, as stated in this way by two participants:

*PT: So, there is something in having hands on and, and we have to, so –*

*OT: We have to.*

*PT: We do, we have our hands on [the infant] when we are out [on home visits]. After all, I can’t advise and guide unless I have my hands on. I simply can’t.*

*OT: I can’t just examine with my eyes.*

*PT: No, and I then have to feel and sense the infant's response.*

*OT: Feel and sense their reactions to sensations.*

*PT: And just how, and it is only then that I can actually coach in the right way in relation to the parents. (Municipality 14)*

To be able to provide early intervention therapy services, participants also found it essential that, through experience, therapists gained competencies in managing the role of a guide for parents/caregivers.

*Institutional challenges to training and specializing.* Participants described that it could be problematic for inexperienced therapists to gain the required experienced-based skills from colleagues due to the organizational structure of many workplaces, where there were only one or few groups of PTs and OTs working with children. Participants mentioned that some workplaces did not have therapists with experience with CP. Another challenge was being up-to-date with new research-based knowledge about CP. In particular, participants working in municipalities experienced difficulty in being specialized in specific populations, such as children with CP, because of a lack of time and the decentralized structure, where children with CP made up only a small percentage of all the children receiving therapy. One participant stated that she sought knowledge and support in cases about CP from specialized therapists at the hospital, who were working on the CPOP program,^25^ as she could not be an expert in CP when she had so many other health conditions to address.

*PT: I cannot be great at this [working with children with CP], when I have so many other tasks. So, when there is something I’m in doubt about, I have always rung and asked [the therapists specialized in CP], and they are both extremely kind to come out [on joint home visits] and help, and to watch, yeah, so I have actually used that very much. (Municipality 20)*

## Discussion

This study explored therapists’ experiences of providing early intervention therapy services for infants with or at risk of CP. The analysis resulted in two overall themes with four corresponding categories and 9 subcategories showing that early therapy services are influenced by a complex practice and a high level of requirements for therapeutic skills. The study findings indicate that there are several challenges in applying EBP, including incorporation of up-to-date research knowledge into clinical practice.

Findings from the present study show that participants emphasized the value of interdisciplinary and cross-sectoral meetings, describing them as constructive spaces for communication, coordination, and mutual understanding. These positive experiences highlighted the potential of such collaboration to support more coherent and family-centered support. In this context, an early inclusion of infants at risk of CP in a follow-up program, such as the Danish CPOP,^25^ could promote collaboration and thereby increase the quality of early services offered to these infants and their families. However, findings indicated that the collaboration between hospitals and municipalities was often hindered by insufficient exchange of professional information between sectors, whereby parents had to convey the information. Lack of cross-sectoral communication may lead to information loss during transitions and affect the application of EBP. The absence of established routines for cross-sectoral communication suggests that therapists and their teams at times operate independently, with minimal communication or coordination with other professionals, rather than engaging in collaborative, cross-sectoral practices aimed at integrated support. The tendency for fragmentation of services across sectors presents a known challenge for service providers and families to overcome within the healthcare system.^29^ In fact, inadequate collaboration and communication between all involved in the infants’ intervention has been reported the main challenge in early intervention therapy services, as perceived by PTs and OTs,^22–24^ which is supported by this study's finding of lack of interdisciplinary knowledge-sharing regarding recommendations for stretching.

This study found inconsistent use of recommended assessment tools for early detection of CP, such as the GMA and HAI, which challenges EBP, as these tools contribute to enabling an early and specific intervention, which is crucial for optimizing infants’ functional outcomes.^7,12^ This inconsistent use may be related to the fact that, historically, there has been a wait-and-see attitude toward the diagnosis of CP.^10,30^ However, low use of the GMA is consistent with findings in studies from other high-income countries.^15,31,32^ Barriers reported include lack of time, funding for courses, formal training and standardized pathways.^15,31,32^ Another finding was that EBP may be challenged by the fact that therapists were not a part of an interdisciplinary program or routine for early detection of risk of CP at the hospitals. The lack of a standard routine can lead to disparities in early detection and diagnosis of CP, which can impact the timely initiation of evidence-based early therapy interventions.^7,10^ Therefore, a pathway for an early interdisciplinary detection of risk of CP needs to be implemented in clinical practice in Denmark, as seen elsewhere.^33–35^ A positive finding was that participants initiated referral for diagnostic evaluation at hospital when they identified motor signs of CP in infants without a medical history indicating risk of CP, which enables early EBP.

Another dilemma in relation to EBP was the finding of a greater focus on promoting infants’ gross motor skills, rather than hand function in early age, which has implications for the incorporation of evidence-based early therapy interventions targeted at hand function. Interventions for hand function were typically offered at a later age, when a child was expected to have the skill. This suggests that the window of opportunity in the first year of life may be missed,^8–10^ potentially limiting optimal hand development and thereby affecting the child's ability to handle toys and participate in play. Play is the primary occupation for infants and children^36^ and a term consistent with participating in activities in the International Classification of Functioning model,^37^ which highlights the importance of early attention to engaging infants in play including providing postural support that allows for the best use of hands^36^ either with manual support or assistive devices. However, the implementation of best research knowledge into clinical practice is recognized as a complex process that depends on several factors, such as organizational culture, management, and the motivation and competencies of the individual health professional.^38^ Studies have shown a positive attitude towards EBP among health professionals, but this attitude was not reflected in their practice because of barriers, such as lack of time and skills,^38,39^ and because activities directly related to maintaining patient flow through the health service were given higher priority by both clinicians and managers.^38^

In accordance with current research evidence, a broad agreement was found among participants that early therapy interventions must be integrated into daily activities and routines, which allows for repetition.^2,14^ Thus, early therapy interventions are dependent on the involvement of parents as treatment providers, which may have an impact on achieving EBP. Parental involvement can be practiced in many ways and with varying degrees of influence on decision making.^40^ It was found that the approaches to involving the parents in the therapy intervention varied from explaining and illustrating exercises to shared goal setting. In this regard, it is important to note that, although goal setting is considered to be a main component of FCP,^18,41^ a family-centered approach supports families’ own wishes for involvement.^40,41^ This supports the importance of therapists being able to enter the role that best meets the current needs of families,^42^ which is especially important in families who are in a new and vulnerable life situation with an infant with or at risk of CP.

Overall, the findings in the present study suggested that providing early intervention therapy services required the therapist to be able to act in a very diverse role as a guide to the parents. This role involved adapting guidance, information and support to parents’ stage of crisis, resources and ability to convert guidance into practice. It also involved navigating between parents’ different attitudes and cultural backgrounds, which aligns with findings from other studies.^19,23,24^ This finding also indicates that the intervention performed is linked to the therapists’ perceptions of the family situation and preferences. A family-centered approach is considered a good way to elicit family preferences relevant to EBP.^43^ However, incorporating the user perspective into EBP is a complicated process that requires professional competencies and, in particular, good communication skills.^44^ According to the Intentional Relationship Model, therapists must be competent in using the following six communication modes to remain family-centered: advocating, collaborating, empathizing, encouraging, instructing and problem-solving.^45^ However, the use of the collaborative mode (involving parents in goal setting) and problem-solving mode (discussing the best possible intervention with the parents) did not emerge clearly from participants’ experiences in the current study. Limited use of these modes may relate to the therapists’ habits or assessment that the parents needed a lower degree of involvement.^42^ However, it can also be an expression of the difficulty of entering into a collaborative relationship because of the requirement of competencies, as is evident from this study's finding that successful goal setting depended on the therapists’ skills. Acknowledging that shared decision making and goal setting may be difficult, Novak et al. have recently proposed a model for decision making within EBP.^20^ This model could serve as a support for therapists in the therapist-family collaboration, in terms of making decisions that best achieve the family's goals, thus promoting the quality of the therapy intervention provided.

The participants’ accounts pointed to a noteworthy subtheme regarding knowledge-seeking parents and the misinformation they often encounter. This underscores the importance of understanding how parents navigate, interpret, and act upon health-related information. As this study did not include first-person perspectives from parents, further research is needed to explore how these processes unfold from the parental point of view.

The current study found the interventiońs reliance on parents as therapy providers may pose a dilemma in meeting infants’ training needs. Sometimes, it was not possible to reach a high training intensity, as supported by evidence,^2,14^ for various reasons. In these cases, participants found that the role of the therapist was to gradually support the parents in becoming more involved. This approach is consistent with FCP; it implies that therapists are aware of not causing additional stress or placing unwanted responsibility on parents to avoid therapy-related stress as an unintended consequence of therapist-family collaboration in high-dose intervention.^46^ The contrary was also experienced: some parents became too much of a training provider, which can also lead to therapy-related stress.^46^ In these cases, the therapist's role was perceived as helping the parents to slow down and prioritize their efforts, to avoid the training having a negative impact on the parent-infant relationship. Participants’ emphasis on supporting the parent-child relationship is a notable finding, as high-dose interventions can contribute to parents encountering a conflict between their role as a parent and as a therapy provider, which has implications for the sustainable parent-child relationship^46^ that is crucial for infants’ development.^47^ Thus, involving parents as training providers adds to the complexity of the therapists’ role as a guide for parents and emphasizes the importance of integrating the perspective of parents in EBP, as it could enable the therapists to create the best opportunities for the infant to receive an evidence-based CP-specific intervention.

The perceptions portrayed in this study were that the necessary therapeutic skills for providing early therapy services to infants with or at risk of CP go far beyond basic therapist education. These skills include clinical expertise in assessing infants’ sensorimotor development and training needs, competencies in guiding parents with complex needs and being specialized in CP. Interestingly, it was found important to sense the infant's response to stimulation with hands as a basis for planning and providing intervention. This can be understood in light of these infants’ lifelong need for external postural support to promote participation^48^ and to reduce secondary impairments. Thus, the provision of early intervention therapy services requires educating and training therapists, a need supported by the literature.^10,19,22–24^ This makes therapist education an essential factor for implementing EBP.

However, this study found that neither being trained by colleagues nor being specialized was supported by a decentralized organization. This finding is consistent with the fact that the organizational structure can constitute a barrier to implementing EBP.^38^ Furthermore, the finding supports the addition of the local context and environment as a source of knowledge in EBP.^44^ Another notable finding was a great emphasis on experience-based knowledge as being fundamental to the provision of a task as complex as an early therapy intervention. Participants’ overall perception was that therapeutic skills should be acquired through training from experienced colleagues, in line with the suggestion by Bowyer et al..^23^ This weighting of practical knowledge is important for EBP, as it ties the sources of knowledge together^44^ and enables therapists to apply research evidence to the individual infant and family.^49^

### Limitations

A limitation of the study may be the fact that the first author (HSP) is familiar with the clinical practice under investigation, as a researcher's pre-understanding can influence the data collection and analysis.^50^ However, HSP has been aware of her pre-understanding and position during all research processes. Furthermore, to enhance credibility, researcher triangulation was used, in which the other authors who do not work in the field of child services were involved in the research processes. AØH and LRM were involved in the development of the interview guide, and all authors were involved in the analysis, whereby HSP's pre-understanding was challenged and actively used throughout the research process and in team reflections. Thus, HSP's pre-understanding can also be seen as a strength in relation to informing an in-depth data collection and analysis.

The study was limited by the lack of detailed information on the training, qualifications, and experience of the participating therapists. These factors may have influenced their perspectives and practices, and including such data could have added depth to the analysis.

This study is based solely on therapists’ perspectives. While this provided in-depth insight into their experiences, the lack of multiple stakeholder views limited opportunities for triangulation. Future studies could benefit from including additional perspectives to strengthen the findings.

Conducted in Denmark, this study acknowledges variations in health services across countries. However, issues like inadequate interdisciplinary and cross-sectoral communication and the need for therapeutic specialization are widely relevant, supporting the findings’ transferability to other settings where early therapy intervention is provided to infants with or at risk of CP.

## Conclusions

This study found that the provision of evidence-based early intervention therapy services for infants with or at risk of CP is influenced by complex practice and high requirement of therapeutic skills. This included factors such as insufficient coordination and communication between hospitals and municipalities, varying use of motor assessment tools to detect risk of CP, more focus on gross motor function than hand function in early age, impact of usual clinical practice, requirements for acting in a variable therapeutic role when providing guidance for parents, and challenges obtaining the necessary therapeutic training and specialization in a decentralized organization. These factors may have an impact on EBP and need to be addressed in order to optimize infants’ outcomes through the incorporation of the best-available research evidence into a family-centered clinical practice.
